# Beyond Earth, Beyond Time: Preserving Female Fertility in Space Missions

**DOI:** 10.3390/jcm14175975

**Published:** 2025-08-24

**Authors:** Loris Marin, Luciana Bordin, Chiara Sabbadin, Guido Ambrosini, Alessandra Andrisani

**Affiliations:** 1 Department of Women’s and Children’s Health, University of Padua, Via Giustiniani 3, 35128 Padua, Italy; luciana.bordin@unipd.it (L.B.); guido.ambrosini@unipd.it (G.A.); alessandra.andrisani@unipd.it (A.A.); 2 Endocrine Unit, University-Hospital of Padova, 35128 Padova, Italy; chiarasabbadin.85@gmail.com

**Keywords:** fertility preservation, fertility counseling, astronauts, space travel, radiation, microgravity

## Abstract

The number of female astronauts participating in space missions is increasing, and concerns about the impact of spaceflight on reproductive health have emerged. Space radiation and microgravity pose potential threats to ovarian reserve and uterine function, but data on human female reproductive health in space remain scarce. This review explores current evidence from both real and simulated space conditions, including animal studies and ground-based cosmic radiation models. The relevant literature on cosmic radiation, fertility preservation strategies, and gynecological risk management in spaceflight was analyzed to provide a comprehensive synthesis. Space radiation might damage ovarian follicles and impair folliculogenesis, potentially leading to premature ovarian failure and microgravity might alter endocrine function. While human data are lacking, murine and in vitro model studies suggest significant reproductive risks. Embryo/oocyte and ovarian tissue cryopreservation are currently the most viable fertility preservation strategies. Shielding technologies, radioprotective agents, and hormonal modulation may offer adjunct protection. In conclusions, fertility counseling and preservation should become integral to pre-mission planning for female astronauts of reproductive age. A personalized approach, accounting for individual reproductive goals, age and mission duration, is essential. Further research is urgently needed to understand the reproductive effects of deep space travel and to develop targeted protective strategies.

## 1. Introduction

The first human flew in space in 1961, and the first woman orbited the Earth in 1963. Despite this early milestone, female participation in space missions remained limited for several decades. Concerns were raised regarding menstruation during space travel, with early beliefs suggesting that menstruating women were more accident-prone, and women were even described as “temperamental psycho-physiologic humans”, deemed unsuited for piloting spacecraft [1].

In recent years, however, there has been a notable and steady increase in the number of female astronauts participating in both short- and long-duration missions [2]. This shift has been driven by deliberate efforts from space agencies such as NASA, ESA, and others to promote gender diversity and inclusion in astronaut selection [2,3]. Among NASA’s upcoming goals it to land the first woman on the lunar surface [4]. However, the efforts to increase female representation in space crews must be supported by adequate scientific knowledge to ensure safe space travel and by legislation that protects women’s health [5,6,7,8]. Understanding the health effects of spaceflight on female astronauts is crucial not only for ensuring safety and performance, but also for the successful planning of long-term missions, including those to Mars and beyond [9,10]. Microgravity and space radiation present significant challenges to human exploration beyond Earth’s protective atmosphere and magnetic field [5,11,12,13]. Assessing the composition, sources, and levels of space radiation is essential to evaluating health risks, particularly to radiation-sensitive organs such as the ovaries. Although the effects of spaceflight on female reproduction health remain largely unknown, astronauts should receive fertility and pregnancy counseling before missions. However, such counseling is complicated by the lack of data on how space conditions affect female reproduction.

## 2. Composition and Sources of Space Radiation

**Figure 1:**
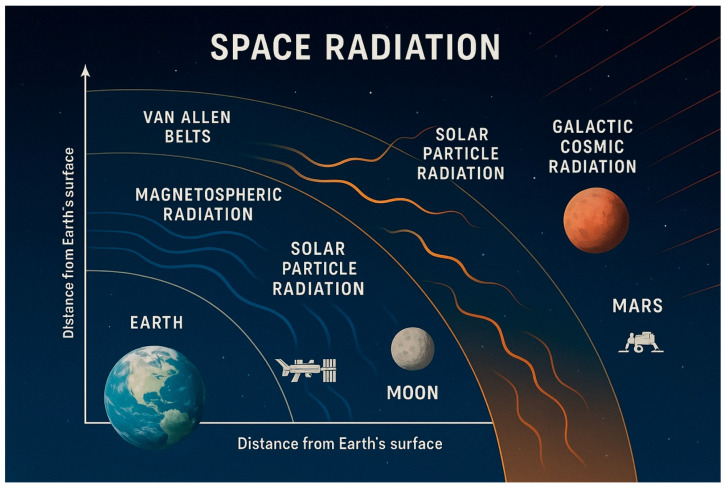
Distribution of space radiation types by distance from earth.

Space radiation consists of three main components: trapped radiation in the Van Allen belts, Solar Particle Events (SPE), and Galactic Cosmic Rays (GCR) [14,15,16]. Each radiation type has distinct spatial distributions and varying relevance depending on mission profiles (Figure 1).

This figure illustrates the spatial distribution of the main types of space radiation—magnetospheric radiation, trapped Van Allen belts, solar particle radiation, and galactic cosmic radiation—relative to increasing distance from Earth. Key mission zones such as Low Earth Orbit, lunar orbit, and Mars transit are indicated to contextualize exposure risks for astronauts.

The Van Allen belts are regions of charged particles, primarily electrons and protons, trapped by Earth’s magnetic field. They are divided into two zones: the inner belt, mainly composed of highly energetic protons (10–400 MeV), and outer belt, primarily consisting of electrons with energies from several keV to several MeV (Table 1). The inner belt poses significant radiation risks for spacecraft operating between 1000 km and 12,000 km, while the outer belt affects geostationary satellites situated between 13,000 km to 60,000 km altitude [14,15,16]. Astronauts aboard the International Space Station (ISS), orbiting at around 400 km, generally avoid significant exposure from these belts. However, missions passing through or operating within them require advanced radiation shielding. Solar Particle Events are unpredictable and sporadic emissions from the Sun, sting hours to days. These events release high-energy protons and other charged particles, with energies ranging from tens to several hundred MeV. Such particles can spacecraft shielding and biological tissues, posing acute radiation risks, especially for missions outside Earth magnetosphere [14,15,16]. Historical events, such as the 1972 solar storm during the Apollo program, highlight the potential danger of SPEs [17] (Table 1).

GCRs originate from outside the solar system, primarily generated by supernovae and other high-energy cosmic events within the Milky Way. GCRs are composed of the following components:

- Protons (85%): High-energy hydrogen nuclei that make up the majority of cosmic radiation.
- Helium nuclei (14%): Energetic alpha particles with strong penetrative capacity.
- Heavy ions (~ 1%): Nuclei of heavier elements (e.g., carbon, oxygen, neon, silicon, iron) with high Linear Energy Transfer (LET), causing severe DNA and cellular damage [14,15,16].

GCR intensity is inversely related to solar activity due to solar wind modulation. Consequently, astronauts face higher GCR exposure during solar minimum periods. Overall, radiation exposure varies significantly based on mission location, duration, and solar cycle (Table 1).

## 3. Radiation Mitigation Strategies in Spacecraft Design

Radiation exposure represents one of the most critical health risks for astronauts. The abovementioned sources of ionizing radiation encompass a wide spectrum of particle types which are capable of inducing various forms of biological damage, including carcinogenesis and neurodegeneration. One strategy to mitigate these harmful effects involves the design of spacecraft shielding systems that provide passive protection against high-energy particles. The goal is to strike an optimal balance between the safety and radioprotective properties of the selected materials and their aerodynamic performance. An effective radiation shielding system should exhibit adaptability, long-term structural integrity, and biocompatibility, while also being cost-effective and minimizing the production of secondary radiation [18]. Aluminum, widely used in aerospace engineering, offers favorable mechanical and aerodynamic properties. However, from a radiobiological perspective, it is suboptimal due to its relatively high atomic number, which promotes the production of secondary particles. These secondary particles can significantly increase the LET profile within the spacecraft, thereby enhancing the biological effectiveness of the radiation. The charge-to-mass ratio (Z/A) is a critical parameter influencing a material’s interaction with ionizing radiation, where Z represents the atomic number (i.e., the number of protons in the nucleus) and A denotes the mass number (i.e., the total number of protons and neutrons) [18]. Materials with a high Z/A ratio are considered for shielding against high-energy particles due to their favorable energy attenuation capabilities; however, they are also associated with increased nuclear fragmentation. In contrast, materials with a low Z/A ratio, such as hydrogen-rich compounds, polyethylene (PE) and other advanced polymers, can reduce the production of secondary particles [18]. For these reasons, the selection of shielding materials should be carefully tailored to the specific characteristics of the radiation environment and the functional requirements of the mission. High-density polyethylene has demonstrated high shielding efficacy and is increasingly regarded as a promising candidate material for habitat modules and personal protective systems. Hydrogen-rich composites, such as boron nitride–polyethylene blends, novel polymeric aerogels and hydrogenated hybrid laminates are also being considered for spacecraft applications, particularly in long-duration missions to Mars and other deep-space destinations, due to their favorable radioprotective properties [19,20]. Polymeric composites, nanocomposites, and multi-layered materials have emerged as lightweight and effective alternatives to traditional shielding solutions [19,20]. Nevertheless, the development of high-performance multilayer shielding systems capable of addressing the complex interplay between material properties and mission-specific requirements remains a substantial engineering and scientific challenge [18]. In addition to passive shielding strategies, active radiation protection is under investigation as a complementary means to reduce astronaut exposure. The application of magnetostatic or electrostatic fields may serve to deflect charged particles in the space environment, functionally replicating the natural protective barrier provided by Earth’s magnetosphere. However, these systems are still at a low technological readiness level and pose significant challenges, including high power demands, system stability, and potential risks to crew health [21].

A NASA report from the 2004 Advanced Radiation Protection Working Group workshop evaluated four primary active shielding concepts, identifying their respective advantages and limitations. Of the four, three were considered unfeasible due to prohibitive requirements in terms of mass, power consumption, and safety [22]. In contrast, the unconfined magnetic field concept was highlighted for further development and study [23]. This approach involves the generation of a strong magnetic field of deflecting both solar and galactic cosmic radiation. The external placement of superconducting magnets has been regarded favorably due to reduced shielding demands and enhanced spatial control of the magnetic field distribution [22]. Spacecraft are also being designed with dedicated radiation storm shelters; small, shielded compartments strategically located within the spacecraft’s interior. These shelters are often constructed by repurposing onboard consumables such as water containers, food packages, or sleeping bags to provide additional protection during acute SPEs, which can occur with minimal warning and reach hazardous dose rates within a matter of hours [24]. Given the need for water during any spaceflight, and considering that water is hydro-gen-rich and composed of low atomic number elements, its use for radiation shielding is among the most commonly proposed strategies in the design of space habitats for crewed missions [25]. Deployable water-based shelters, particularly those incorporating water walls, are most suitable for short- to medium-duration missions involving small crews. These systems require rapid water transfer capabilities to allow simultaneous wall deployment and drainage. Their feasibility, however, is highly dependent on available internal volume, the spatial configuration of onboard systems, and the flexibility to rearrange interior elements to accommodate the shelter infrastructure. For longer-duration missions involving larger crews, a more permanent solution may involve the integration of water bladders directly into the structural walls of the habitat. While this approach enhances shielding continuity, it also introduces significant mass penalties due to the large water volumes required, which may pose challenges for compliance with launch mass limitations [25]. Mission parameters, including duration, crew size, launch capacity, and both structural and operational configuration of the habitat, must be carefully considered when selecting and integrating radiation shielding solutions.

## 4. Cosmic Radiation Effects on Human Health

Prolonged exposure to cosmic radiation during spaceflight poses significant health risks, particularly during missions beyond low Earth orbit, where the protective effect of Earth’s magnetic field is greatly diminished. Space radiation can cause dense ionization tracks and clustered DNA damage that are difficult for cellular repair systems to manage [26,27]. The health effects of such exposure extend across multiple physiological systems, with both acute and long-term clinical implications, in particular the visual system, skeletal integrity, and the increased likelihood of carcinogenesis.

Ocular effects, such as radiation-induced cataract formation, have been well-documented in animal models and astronauts, especially those on extended missions aboard the ISS. The lens of the eye is particularly sensitive to ionizing radiation, with posterior subcapsular opacities being a common early manifestation [28]. Importantly, cataract genesis is a deterministic effect, meaning it occurs above a threshold dose, but that threshold may be lower in the context of repeated low-dose exposure. Another prevalent phenomenon is the Spaceflight-Associated Neuro-ocular Syndrome (SANS), characterized by optic disc edema, globe flattening, choroidal folds, and a posterior shift in the brain [29,30]. While the etiology of SANS is multifactorial and involves fluid redistribution in microgravity, ionizing radiation has been proposed as a contributing factor. Cosmic rays may induce oxidative stress and damage retinal and optic nerve tissues, potentially exacerbating or accelerating neuro-ocular degeneration [29,30]. Animal models exposed to heavy ions have shown retinal cell death and vascular degeneration, supporting the hypothesis that high-LET radiation plays a role in spaceflight-induced visual impairment [31]. Another system vulnerable to space radiation is the central nervous system [32]. Unlike somatic tissues, the central nervous system exhibits limited regenerative capacity, making it particularly susceptible to long-term damage from ionizing radiation. Experimental evidence, primarily from rodent studies exposed to simulated galactic cosmic radiation, has demonstrated a range of deleterious effects, including impaired neurogenesis, cognitive decline, and neuroinflammation, and oxidative stress [33,34,35]. These alterations are associated with significant cognitive deficits, such as impairments in spatial learning, memory retention, executive function, and sensorimotor coordination [33,34,35]. Of particular concern is the dose-dependent nature of these effects. Even low doses of high-LET particles can disrupt neural networks and neuroplasticity [36,37]. Such changes may persist long after exposure, indicating a potential for chronic or progressive CNS dysfunction [37,38]. While extrapolation to humans remains uncertain, these findings raise concerns for long-term neurological outcomes, including neurodegeneration. Moreover, emerging evidence suggests that cumulative radiation exposure could elevate the risk of long-term neurodegenerative disorders, including Alzheimer’s disease–like pathology and accelerated brain aging [39]. The hematopoietic and immune systems are also at risk from space radiation exposure. Ionizing radiation is known to suppress bone marrow hematopoiesis, reduce lymphocyte counts, and impair the functionality of immune cells such as T-lymphocytes and natural killer cells [40]. These effects may lead to immunosuppression, thereby increasing the crew’s susceptibility to infections and diminishing immune surveillance against pre-neoplastic and neoplastic cells, potentially heightening the risk of tumor development during or after long-duration missions [40,41]. In addition to hematopoietic concerns, skeletal demineralization and musculoskeletal deterioration represent other critical challenges. While microgravity alone induces significant bone loss by promoting osteoclastic bone resorption and inhibiting osteoblastic bone formation, particularly in weight-bearing skeletal regions such as the pelvis, femur, and lumbar spine, radiation exposure appears to exacerbate this phenomenon [42,43]. In fact, radiation has been shown to impair osteoblast function and stimulate osteoclast activity, leading to net bone loss [42,43]. Studies in rodent models exposed to simulated galactic cosmic radiation have demonstrated deterioration in microarchitectural parameters (e.g., trabecular number and thickness), and increased skeletal fragility, delayed fracture healing, and impaired functional recovery post-mission [42,43,44]. The combined effects of microgravity and radiation present a synergistic threat, whereby the skeletal system may suffer more profound damage than would be expected from either factor independently. One of the most concerning biological effects of cosmic radiation is the increased risk of carcinogenesis. Cosmic radiation includes high-energy protons and heavy ions, capable of producing complex DNA damage that is difficult to repair. This damage includes clustered double-strand breaks, chromosomal aberrations, and persistent oxidative stress, all of which contribute to genomic instability and are significantly more difficult to repair compared to isolated lesions typically caused by low-LET radiation [45,46]. Epidemiological models suggest elevated lifetime cancer risks for astronauts on extended missions. This risk is modulated by factors such as age at exposure, sex, genetic susceptibility, tissue radiosensitivity, and radiation quality [26,41]. Particular attention has been drawn to female astronauts, who may exhibit increased radiosensitivity in organs such as the breast, thyroid, and reproductive tissues [46,47,48,49]. These findings emphasize the importance of sex-specific risk modeling and targeted shielding strategies to protect radiosensitive tissues during exploration-class missions. Significant uncertainty remains in predicting cancer incidence due to limited availability of human data on space-relevant exposures. This necessitates the ongoing refinement of radiobiological models, in vitro studies using human tissue analogs, and advanced computational dosimetry to support future mission planning to the Moon, Mars, and beyond.

## 5. Studies on Cosmic Radiation on Reproductive Health

The complex mixture of space radiation is considered particularly concerning due to its significant biological effects, especially its carcinogenic potential [5,16,49]. Given the challenges of conducting in vivo studies in space, most investigations rely on ground-based simulations using particle accelerators [6,11]. The NASA Space Radiation Laboratory (NSRL) is designed to replicate key components of the space radiation spectrum, including protons and high atomic number and energy (HZE) ions such as ^56^Fe and ^28^Si. These facilities offer precise control over radiation dose, type, energy, and timing, enabling detailed mechanistic studies in animal models and cell systems [45]. NSRL-based studies are valuable for simulating mixed-field exposures with consistent and reproducible beam profiles [45]. However, limitations include the absence of microgravity, the inability to perform human studies, and a focus primarily on acute rather than chronic low-dose exposures. Reproductive endpoints have been investigated in invertebrates, amphibians and fish after exposure in low Earth orbit [50,51,52], while murine models have been studied under Earth-based simulated conditions [53,54]. Regarding male fertility, several animal studies have shown that exposure to ionizing radiation, especially high-LET particles, can significantly impair male reproductive function. In rodent models, such exposure leads to testicular atrophy, reduced sperm count and motility, disrupted spermatogenesis, and increased germ cell apoptosis [55,56]. Human data on this topic are limited, yet suggestive. Evidence from the Literature indicates that both microgravity and ionizing radiation negatively affect human male reproductive parameters [56,57]. Specifically, sperm motility was impaired by both factors, while a reduction in total sperm count appeared to be associated with microgravity alone [56]. Additionally, both stressors contributed to increased sperm DNA fragmentation. Microgravity also led to declines in testosterone levels and testicular mass [56]. Moreover, changes in telomere length and gene regulation impairing male fertility were also reported [57]. It is crucial to not only investigate the effects of space radiation on male fertility, but also to develop and evaluate effective strategies for mitigation and protection.

Regarding female fertility, only a few studies have addressed reproductive health specifically under simulated cosmic conditions. Evidence suggests that space conditions impair folliculogenesis. Disruption of estrous cycle during spaceflight has been observed [58], along with compromised in vitro follicle development in mice [59]. Mishra et al. demonstrated that irradiating adult female mice with HZE iron particles induces DNA double-strand breaks, oxidative damage, and apoptosis in ovarian follicles, resulting in premature ovarian failure [49,60,61]. The authors further reported that exposure to charged oxygen particles resulted in even greater follicular depletion compared to iron ions, suggesting that lighter ions with different LET properties may exert comparable or even enhanced gonadotoxic effects [60,61]. These findings raise substantial concern for female astronauts who, during space travel, may be simultaneously exposed to a complex spectrum of high-energy charged particles, including both iron and oxygen ions. The potential protective role of alpha-lipoic acid (ALA), an antioxidant supplementation, has been investigated as a countermeasure against radiation-induced ovarian damage [60,61]. While ALA supplementation provided partial protection by attenuating certain markers of oxidative damage and apoptosis, it failed to prevent the eventual depletion of ovarian follicles [60,61]. These results highlight the complexity of space radiation–induced ovarian toxicity. Future research is essential, and NSRL-based studies could prove valuable due to their high level of control over radiation parameters. However, it is important to recognize that murine ovarian tissue differs significantly from human tissue. Further studies using other animal models–such as sheep, whose ovarian tissue closely resembles that of humans–are needed [62].

Most space missions to date have occurred in low Earth orbit, where radiation exposures is significantly lower compared to deep space. Consequently, the impact of cosmic radiation on reproductive health during short-duration missions, including space tourism, is likely minimal (Table S1). However, upcoming projects such as NASA planned lunar space station will dramatically increase the number of missions into deep space. Alongside increased lunar travel, human missions to Mars are a longstanding goal of astronautics and they are becoming increasingly feasible, raising additional concerns for long-duration exposure.

Although most missions have remained in low Earth orbit, the Apollo program was the first to operate in deep space, successfully landing humans on the Moon. It also generated important data on radiation exposure in that environment, estimating daily exposure levels of 0.5–1 mSv/day, with cumulative doses between 5 and 10 mSv due to the relatively short mission durations (up to weeks) [16,63]. Another relevant mission is Artemis I, which includes the BioSentinel spacecraft, an astrobiology experiment launched to operate in the deep space radiation environment [64]. The goals is to detect, measure, and compare the impact of deep space radiation on DNA repair over time. Two identical BioSentinel payloads were developed: one for the ISS, which offers similar microgravity but lower radiation exposure, and another as a delayed-synchronous ground control at Earth gravity and radiation levels. This dual setup enables a precise comparison of biological impact of radiation at different distances from the Earth.

In addition to the ovaries, the uterus may also be affected by radiation exposure. Studies on women treated with pelvic radiotherapy show that high doses (40–65 Gy) of low LET radiation lead to decreased uterine size and thickness on imaging, with histological evidence of endometrial and myometrial atrophy [65,66]. The damage is more pronounced in individuals treated at a younger age; even doses between 9 and 54 Gy in prepubertal girls have resulted in significant uterine shrinkage. While uterine damage from radiation appears irreversible when exposure occurs before puberty, estradiol therapy has been shown to restore uterine volume, endometrial thickness and blood flow [65,66].

Further studies are needed to evaluate the effects of charged particle radiation and prolonged microgravity on uterine tissue in spaceflight conditions. Magnetic Resonance Imaging (MRI) with contrast-enhanced perfusion techniques offers a non-invasive and reproducible method to evaluate endometrial and myometrial blood flow, vascular density, and tissue viability. In terrestrial settings, pelvic MRI has already been employed to characterize uterine perfusion in oncological and fertility-preserving contexts, including in women exposed to radiotherapy. A proposed experimental model could involve the use of animal surrogates exposed to simulated cosmic radiation followed by serial MRI evaluations of uterine vascular architecture. Functional imaging outcomes could be correlated with histological and molecular markers of radiation-induced tissue damage, including fibrosis, endothelial dysfunction, and hormonal responsiveness. Ultimately, integrating advanced imaging into radiobiological studies may not only enable early detection of subclinical uterine injury but also inform the development of targeted countermeasures, such as pharmacologic vasoprotective agents or hormonal modulation, to preserve uterine function in female astronauts during and after long-duration space missions.

## 6. Fertility Counseling for Female Astronauts

**Figure 2:**
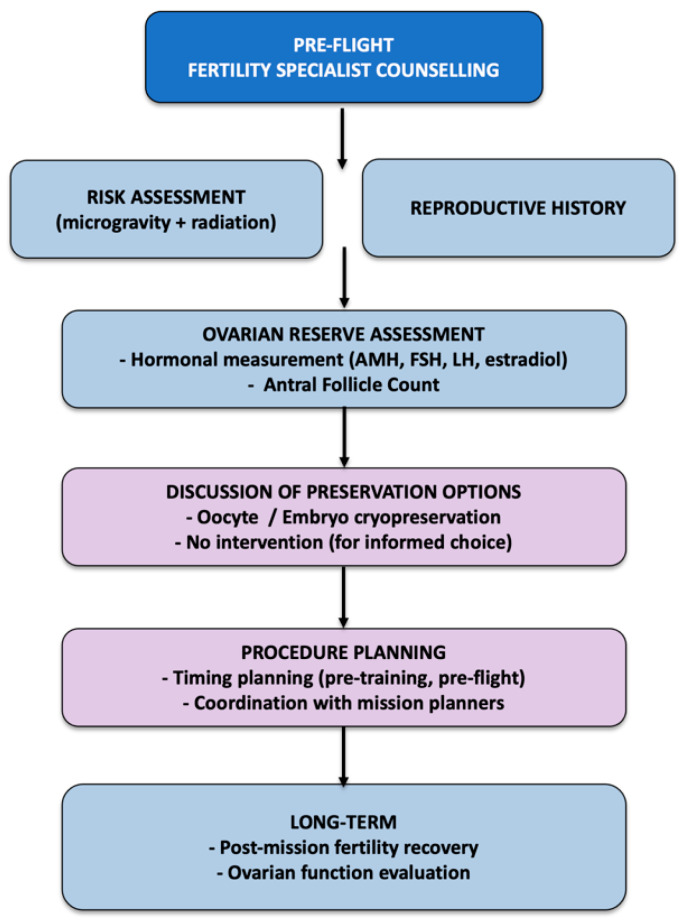
Flow chart fertility preservation counseling for female astronauts.

Female astronauts preparing for space missions may face several challenges related to fertility and family planning. First, many may choose to delay childbearing until spaceflight, but this postponement can significantly impact ovarian aging and reproductive potential. It has been estimated that the combined time required for candidate selection (≈2 years), astronaut training (≈2 years), mission assignment (2–5 years), mission-specific preparation (≈1.5 years), and actual flight (0.5–3 years) could extend the period of menstrual suppression to approximately 11 years [9]. Second, there are no human data on the effects of long-duration deep space missions on fertility. However, existing evidence suggests that microgravity and cosmic radiation could adversely affect female reproductive function [11,16,59]. In one murine study, disruption of estrous cycle and impaired folliculogenesis were observed during spaceflight [60]. To manage menstruation, astronauts often use combined estrogen-progestin pills, which can also help prevent bone loss due to estrogen deprivation [9,12]. Given the potential for prolonged space conditions to compromise ovarian reserve and endocrine function, fertility counseling before and after space missions is becoming an essential component of astronaut healthcare (Figure 2) and it should involve collaboration among reproductive endocrinologists, space medicine specialists, radiobiologists, and psychologists.

Fertility counseling should be integrated into pre-mission medical and psychological evaluations. Discussions should include reproductive planning, the age-related decline in fertility, and the compounded effect of delaying pregnancy due to mission schedules [67]. Not only the duration of the mission but also the age at the departure must be considered. Currently, there are no data on how age may influence the impact of cosmic environments on ovarian reserve and uterine function.

Women should also receive guidance regarding menstrual management in space. They must be informed about the possibility of menstruating during the mission, as well as the practical considerations related to sanitary product use in microgravity. Cycle irregularities may also occur due to stress and microgravity-induced endocrine dysregulation [9,16,58]. Alterations in luteinizing hormone (LH) and follicle-stimulating hormone (FSH) levels have been reported in space, though their clinical relevance remains uncertain [54,60]. For short missions (e.g., two weeks), menstrual cycles can be scheduled or suppressed using contraceptive pills. However, for long-duration missions, astronauts face a logistical dilemma: carrying hundreds (or even thousands) of contraceptive pills or bringing an adequate supply of tampons; neither of which is ideal. Additionally, urine recycling system on ISS were not designed to process menstrual blood, creating further operational concerns [9]. An alternative is the use of long-acting reversible contraception (LARC), such as intrauterine devices (IUDs) or progestin implants.

Although direct human data on DNA damage and oxidative stress from GCRs and SPEs are limited, the risk of accelerated depletion of ovarian follicles due to high-LET radiation remains biologically plausible [16,45,61,68]. This uncertainty should be addressed transparently in pre-mission counseling to empower astronauts to make informed choices about their reproductive future.

A comprehensive fertility counseling session should encompass a detailed reproductive history, including menstrual cycle regularity, previous pregnancies, fertility treatment history (if any), hormonal contraceptive use, and known gynecological or genetic disorders affecting fertility. Hormonal assessment, including anti-Müllerian hormone (AMH) and FSH levels, should be obtained to estimate baseline ovarian reserve [69]. Pelvic ultrasound can further assess antral follicle count and uterine morphology [69]. Once risk stratification is complete, the physician should present evidence-based fertility preservation options, individualized to mission timelines. The emotional and ethical dimensions of reproductive risk and preservation should not be overlooked; mental health support must be embedded in the process [70]. Finally, female astronauts should be informed about the uncertain effects of space radiation on uterine health, particularly during long-term deep space missions. Radiation-induced impairment of uterine vascularization may lead to fibrosis and serious pregnancy complications, including implantation failure, placental disorders, preeclampsia, intrauterine growth restriction, and preterm deliveries [71,72,73]. Future research should aim to establish standardized guidelines for fertility preservation in spaceflight medicine.

## 7. Fertility Preservation for Female Astronauts

Female astronauts may face infertility due to both ovarian and uterine factors arising from multiple space-related stressors. To date, protective strategies such as physical shielding, pharmacological interventions, and operational countermeasures are the only available means to reduce uterine damage during space missions.

Optimizing spacecraft shielding is critical to protecting astronaut health. Advances include the use of hydrogen-rich materials or composite structures designed to absorb or deflect radiation [16,45]. Polyethylene, for example, is highly effective due to its high hydrogen content, which helps minimize secondary radiation generated by high-energy particle interactions. Existing onboard materials, such as water tanks, can also be strategically positioned around crew habitats to enhance radiation protection without adding extra mass [16,45]. Electromagnetic field technologies may eventually deflect charged particles, although this remains under development.

There is increasing interest in radioprotective agents that mitigate ionizing radiation-induced damage. However, no long-term clinical trials have demonstrated their efficacy in preventing the stochastic effects of radiation. Some agents, including metformin and propofol, are already available and have shown radioprotective potential, but additional compounds require investigation through well-designed clinical trials to determine appropriate dosages and side effect profiles [74]. Phytonutrients, which can be obtained with a balanced diet, may also act as radioprotectors due to their antioxidant properties [74,75,76]. Substances like resveratrol, astaxanthin and caffeic acid are already used in clinical contexts to support fertility by reducing inflammation [74,75,76]. Further research is needed to assess the true efficacy of these agents in protecting reproductive tissues during periods of high radiation exposure, ideally with minimal side effects. Space mission planning involves numerous variables, all radiation exposure timing should be considered, especially during periods of increased solar activity or SPEs. While all these countermeasures may contribute to protecting the ovaries and indirectly preserve fertility, established fertility preservation techniques should also be offered to female astronauts [77,78]. The current gold standard includes oocyte/embryo cryopreservation [77,78], depending on partner status and local legislation. In particular, oocyte cryopreservation is a well-established method of fertility preservation that typically entails 10 to 14 days of controlled ovarian hyperstimulation followed by retrieval and vitrification of mature oocytes for potential future use. This approach is especially suitable for individuals seeking to preserve reproductive autonomy and delay childbearing without requiring a partner [79,80]. Embryo cryopreservation offers an alternative for women who have a reproductive partner or intend to use donor sperm. In this case, retrieved oocytes are fertilized in vitro prior to cryopreservation, allowing for the storage of embryos at various stages of development. This option may provide higher success rates upon future use but involves more complex ethical and legal considerations [79,80]. Ovarian tissue cryopreservation is an emerging and innovative fertility preservation strategy, involving the surgical removal and cryogenic storage of ovarian cortical tissue, which harbors a high density of primordial follicles [81,82]. Following mission completion or when fertility restoration is desired, the frozen tissue can be autotransplanted or used in research settings for in vitro folliculogenesis, a technique under active development [82]. However, ovarian tissue cryopreservation may offer the most versatile option for astronauts [81,83]. In cases of premature ovarian insufficiency, ovarian tissue transplantation (OTT) can restore both fertility, with the possibility of spontaneous conception after orthotopic OTT [84], and endocrine ovarian function [81]. Hormonal restoration may be especially beneficial in reducing the risk of disuse osteoporosis, a well-documented issue in astronauts due to the lack of gravitational load. Bone loss averages 1–2% per month, varies by skeletal region, and recovers more slowly than it is lost [8,12]. Reestablishing ovarian hormonal function after menopause or induced hypogonadism may help mitigate fracture risk, even if peak bone mass remains lower [81].

Although ovarian tissue cryopreservation is no longer considered experimental [77], its use for elective purposes remains controversial. In such cases, the anticipated extension in fertility must at least equal the ovarian tissue loss incurred during retrieval. [81]. To evaluate the suitability of ovarian tissue cryopreservation for astronauts, further studies are needed to clarify the extent of ovarian damage caused by cosmic radiation. Ultimately, fertility preservation strategies should be individualized, taking into account the woman’s age, reproductive goals, mission characteristics, and associated risks. If uterine vascularization is significantly impaired during deep space travel, current therapies are unlikely to reverse the damage, potentially leading to irreversible infertility. In such cases, gestational surrogacy may be the only viable solution, where permitted by national legislation.

## 8. Future Studies

Given the current state of knowledge, the conclusions drawn in this review must be interpreted with caution. While the existing body of literature provides preliminary evidence regarding the detrimental effects of space radiation on reproductive health, many of the findings remain probabilistic in nature due to the limited availability of human data and the predominance of animal or in vitro models. As long-duration space missions to the Moon, Mars, and beyond become increasingly viable, the biological impact of spaceflight on female reproductive health requires greater attention. Future studies must adopt a multidisciplinary approach, integrating reproductive biology, radiation physics, and aerospace medicine. Key areas of focus should include: radiation dosimetry specific to ovarian tissue, the development of shielding models to estimate cumulative ovarian exposure, the use of 3D organ-on-chip or ex vivo ovary culture systems to study dose–response relationships and the deployment of animal models, such as mice or sheep, in low-Earth orbit or lunar analog missions to monitor ovarian damage in real-time. Such knowledge could guide effective pre- and post-mission counseling, helping astronauts make informed decisions regarding fertility and long-term hormonal health and supporting selection of the most appropriate fertility preservation technique. Moreover, longitudinal studies should investigate the potential transgenerational effects of radiation-induced reproductive damage.

In conclusion, as space agencies and commercial entities increase female participation in long duration spaceflight, integrating fertility preservation into mission is becoming increasingly important. Fertility counseling and ovarian reserve assessment should be routinely offered to women of reproductive age selected for space travel. The goal of counseling is twofold: to inform astronauts of the potential reproductive risks associated with spaceflight and to empower them to make well-informed decisions regarding fertility preservation options. Given the current limitations in scientific knowledge, these uncertainties must be clearly communicated, particularly astronauts embarking on extended deep space missions.

## Figures and Tables

**Table 1:** Space radiation profiles and associated mission risks.

Radiation Type	Distance from Earth	Main Particles	Exposed Missions	Biological Concern
Van Allen Belts	1000–60,000 km	Protons, electrons	GEO satellites, Apollo, Artemis (transit)	Acute/prolonged exposure risk
Solar Particle Events	>60,000 km and interplanetary	High-energy protons	Moon, Mars, deep-space missions	Acute, unpredictable exposure
Galactic Cosmic Rays	>60,000 km to interstellar space	Protons, heavy ions	All beyond LEO missions (ISS partially)	Chronic exposure, high LET damage

## Data Availability

No new data were created or analyzed in this study. Data sharing is not applicable to this article.

## Supplementary Materials

The following supporting information can be downloaded at: https://www.mdpi.com/article/10.3390/jcm14175975/s1, Table S1: Space Radiation Profiles and Associated Mission Risks.

## Abbreviations

The following abbreviations are used in this manuscript:

SPE	Solar Particle Events
GCR	Galactic Cosmic Ray
LET	Linear Energy Transfer
NSRL	NASA Space Radiation Laboratory
HZE	High atomic number and energy
OTT	Ovarian tissue transplantation
