# Supplementary material for: Beyond Earth, Beyond Time: Preserving Female Fertility in Space Missions
Source: J Clin Med. 2025 Aug 24;14(17):5975. doi: 10.3390/jcm14175975 (PMC12429734; doi:10.3390/jcm14175975)
Supplement: Supplementary file 1 [file jcm-14-05975-s001.zip › jcm-3773747-supplementary.pdf]

Table S1: Space Radiation Profiles and Associated Mission Risks.

Name	Country	Missions	Launch date	Age at Launch	time in space	Max Distance from Earth (km)
VT	Soviet Union	Vostok 6	June 16, 1963	26	2 days, 22 hours, 50 minutes	231
SS	Soviet Union	Soyuz T-5	Jul. 19, 1982	34	19 days, 17 hours, 6 minutes	218
		Soyuz T-12	Jul. 17, 1984	36		
SR	USA	STS-7	Jun. 18, 1983	32	14 days, 7 hours, 46 minutes	307
		STS-41-G	Oct. 5, 1984	33		
JR	USA	STS-41-D	Aug. 30, 1984	35	6 days, 0 hours, 56 minutes	352
		STS-51-L	Jan. 28, 1986	37		
KDS	USA	STS-41-G	Oct. 5, 1984	33	22 days, 4 hours, 49 minutes	352
		STS-31	April 24, 1990	39		
		STS-45	March 24, 1992	41		
ALF	USA	STS-51-A	Nov. 8, 1984	35	7 days, 23 hours, 45 minutes	352
MRS	USA	STS-51-D	Apr. 12, 1985	38	30 days	352
		STS-40	Jun. 5, 1991	44		
		STS-58	Oct. 18, 1993	46		
SL	USA	STS-51-G	Jun. 17, 1985	42	223 days, 2 hours and 50 minutes	400
		STS-34	Oct. 18, 1989	46		
		STS-43	Aug. 2, 1991	48		
		STS-58	Oct. 18, 1993	50		
		STS-76/79	Mar. 22, 1996	53		
BJD	USA	STS-61-A	Oct. 30, 1985	36	50 days, 8 hours, 24 minutes	400
		STS-32	Jan. 9, 1990	41		
		STS-50	Jun. 25, 1992	43		
		STS-71	Jun. 27, 1995	46		
		STS-89	Jan. 22, 1998	49		
MLC	USA	STS-61-B	Nov. 26, 1985	38	10 days, 22 hours, 2 minutes	352
		STS-30	May 4, 1989	42		
ESB	USA	STS-34	Oct. 18, 1989	36	28 days, 14 hours, 6 minutes	400

		STS-50	Jun. 25, 1992	39		
		STS-71	Jun. 27, 1995	42		
KCT	USA	STS-33	Nov. 22, 1989	37	40 days, 15 hours, 45 minutes	400
		STS-49	May 7, 1992	40		
		STS-61	Dec. 2, 1993	41		
		STS-73	Oct. 20, 1995	43		
MI	USA	STS-32	Jan. 9, 1990	39	55 days, 21 hours, 48 minutes	400
		STS-46	Jul. 31, 1992	41		
		STS-62	Mar. 4, 1994	43		
		STS-81	Jan. 12, 1997	46		
		STS-98	Feb. 7, 2001	50		
LMG	USA	STS-37	Apr. 5, 1991	39	38 days, 6 hours, 13 minutes	400
		STS-59	Apr. 9, 1994	42		
		STS-76	Mar. 22, 1996	44		
		STS-108	Dec. 5, 2001	49		
HS	UK	Soyuz TM-12/TM-11	May 18, 1991	28	7 days, 21 hours, 13 minutes	400
TEJ	USA	STS-40	Jun. 5, 1991	32	63 days, 1 hour, 24 minutes	400
		STS-52	Oct. 22, 1992	33		
		STS-67	Mar. 2, 1995	36		
		STS-80	Nov. 19, 1996	37		
		STS-96	May 27, 1999	40		
MHF	USA	STS-40	Jun. 5, 1991	46	9 days, 2 hours, 14 minutes	400
RB	Canada	STS-42	Jan. 22, 1992	47	8 days, 1 hour, 14 minutes	400
NJD	USA	STS-47	Sep. 12, 1992	39	28 days, 1 hour, 5 minutes	400
		STS-60	Feb. 3, 1994	41		
		STS-85	Aug. 7, 1997	44		
MJ	USA	STS-47	Sep. 12, 1992	36	7 days, 22 hours, 30 minutes	400
SJH	USA	STS-54	Jan. 13, 1993	35	210 days, 23 hours, 5 minutes	400
		STS-64	Sep. 9, 1994	36		
		STS-78	Jun. 20, 1996	38		
		STS-101	May 19, 2000	42		

		STS-102/105	Mar. 8, 2001	43		
EO	USA	STS-56	Apr. 8, 1993	35	41 days, 19 hours, 35 minutes	400
		STS-66	Nov. 3, 1994	36		
		STS-96	May 27, 1999	41		
		STS-110	Apr. 8, 2002	44		
JEV	USA	STS-57	Jun. 21, 1993	37	49 days, 3 hours, 49 minutes	400
		STS-63	Feb. 3, 1995	39		
		STS-83	Apr. 4, 1997	41		
		STS-94	Jul. 1, 1997	41		
		STS-99	Feb. 11, 2000	44		
NJC	USA	STS-57	Jun. 21, 1993	35	41 days, 15 hours, 32 minutes	400
		STS-70	Jul. 13, 1995	37		
		STS-88	Dec. 4, 1998	40		
		STS-109	Mar. 1, 2002	44		
CM	Japan	STS-65	Jul. 8, 1994	42	23 days, 15 hours, 39 minutes	400
		STS-95	Oct. 29, 1998	46		
YVK	Russia	Soyuz TM-20	Oct. 3, 1994	37	178 days, 0 hours, 0 minutes	400
		STS-84	May 15, 1997	40		
EC	USA	STS-63	Feb. 3, 1995	39	36 days, 7 hours, 11 minutes	400
		STS-84	May 15, 1997	41		
		STS-93	Jul. 23, 1999	43		
		STS-114	Jul. 26, 2005	49		
WBL	USA	STS-67	Mar. 2, 1995	36	51 days, 3 hours, 56 minutes	400
		STS-86	Sep. 25, 1997	38		
		STS-91	Jun. 2, 1998	39		
		STS-114	Jul. 26, 2005	46		
MEW	USA	STS-70	Jul. 13, 1995	33	18 days, 18 hours, 30 minutes	400
		STS-101	May 19, 2000	38		
CC	USA	STS-73	Oct. 20, 1995	35	180 days, 3 hours, 0 minutes	400
		STS-93	Jul. 23, 1999	39		
		Soyuz TMA-20	Dec. 15, 2010	50		

CH	France	Soyuz TM-24/TM-23<br>Soyuz TM-33/32	Aug. 17, 1996<br>Oct. 21, 2001	39<br>44	25 days, 14 hours, 22 minutes	400
SSK	USA	STS-83<br>STS-94	Apr. 4, 1997<br>Jul. 1, 1997	36<br>36	19 days, 16 hours, 45 minutes	400
KC	USA	STS-87<br>STS-107	Nov. 19, 1997<br>Jan. 16, 2003	36<br>42	31 days, 14 hours, 54 minutes	400
KPH	USA	STS-90<br>STS-130	Apr. 17, 1998<br>Feb. 8, 2010	39<br>51	29 days, 15 hours, 59 minutes	400
JLK	USA	STS-91<br>STS-99<br>STS-104	Jun. 2, 1998<br>Feb. 11, 2000<br>Jul. 12, 2001	39<br>41<br>42	33 days, 20 hours, 8 minutes	400
JP	Canada	STS-96<br>STS-127	May 27, 1999<br>Jul. 15, 2009	36<br>46	25 days, 11 hours, 57 minutes	400
PM	USA	STS-92<br>STS-112<br>STS-120	Oct. 11, 2000<br>Oct. 7, 2002<br>Oct. 23, 2007	39<br>41<br>46	38 days, 20 hours, 6 minutes	400
PW	USA	STS-111/113<br>Soyuz TMA-11<br>Soyuz MS-03/04<br>Axiom Mission 2	Jun. 5, 2002<br>Oct. 10, 2007<br>Nov. 17, 2016<br>May 21, 2023	42<br>47<br>56<br>63	675 days, 4 hours, 5 minutes	400
SM	USA	STS-112<br>STS-126/119<br>STS-135	Oct. 7, 2002<br>Nov. 14, 2008<br>Jul. 8, 2011	38<br>44<br>47	157 days, 8 hours, 42 minutes	400
LBC	USA	STS-107	Jan. 16, 2003	42	15 days, 22 hours, 20 minutes	400
SW	USA	STS-121<br>STS-120<br>STS-131	Jul. 4, 2006<br>Oct. 23, 2007<br>Apr. 5, 2010	40<br>41<br>44	42 days, 23 hours, 46 minutes	400
LN	USA	STS-121	Jul. 4, 2006	43	12 days, 18 hours, 37 minutes	400
HSP	USA	STS-115<br>STS-126	Sep. 9, 2006<br>Nov. 14, 2008	43<br>45	27 days, 15 hours, 36 minutes	400

AA	Iran / USA	Soyuz TMA-9/8	Sep. 18, 2006	40	10 days, 21 hours, 5 minutes	400
SW	USA	STS-116/117<br>Soyuz TMA-05M<br>Starliner Crewed Flight Test	Dec. 9, 2006<br>July 15, 2012<br>June 5, 2024	41<br>47<br>59	608 days, 19 minutes	400
JH	USA	STS-116	Dec. 9, 2006	42	12 days, 18 hours, 37 minutes	400
TCD	USA	STS-118<br>Soyuz TMA-18<br>Soyuz MS-25	Aug. 8, 2007<br>Apr. 2, 2010<br>Mar. 23, 2024	38<br>41<br>55	372 days, 18 hours, 36 minutes	400
BM	USA	STS-118	Aug. 8, 2007	56	12 days, 17 hours, 53 minutes	400
YSY	Republic of Korea	Soyuz TMA-12	Apr. 8, 2008	30	10 days, 21 hours, 7 minutes	400
KLN	USA	STS-124<br>Soyuz TMA-09M	May 31, 2008<br>May 28, 2013	39<br>44	180 days, 0 hours, 29 minutes	400
LMMA	USA	STS-125<br>SpaceX Crew-2	May 11, 2009<br>Apr. 23, 2021	38<br>50	212 days, 15 hours, ,21 minutes	400
NPS	USA	STS-128/129<br>STS-133	Aug. 28, 2009<br>Feb. 24, 2011	47<br>49	104 days, 23 hours, 38 minutes	400
DML	USA	STS-131	Apr. 5, 2010	35	15 days, 2 hours, 47 minutes	400
NY	Japan	STS-131	Apr. 5, 2010	40	15 days, 2 hours, 47 minutes	400
SW	USA	Soyuz TMA-19<br>SpaceX Crew-1	Jun. 15, 2010<br>Nov. 15, 2020	45<br>55	330 days, 13 hours, 7 minutes	400
LY	China	Shenzhou 9<br>Shenzhou 14	Jun. 16, 2012<br>Jun. 5, 2022	34<br>44	195 days, 0 hours and 50 minutes	400
WY	China	Shenzhou 10<br>Shenzhou 13	Jun. 11, 2013<br>Oct. 15, 2021	33<br>41	197 giorni, 0 ore, 1 minuto	400
YS	Russia	Soyuz TMA-14M	Sep. 25, 2014	38	167 days, 5 hours, 46 minutes	400
SC	ITA	Soyuz TMA-15M<br>SpaceX Crew-4	Nov. 23, 2014<br>Apr. 27, 2022	37<br>45	370 days, 5 hours, 45 minutes	400
KR	USA	Soyuz MS-01<br>Soyuz MS-17	Jul. 6, 2016<br>Oct. 14, 2020	38<br>42	300 days, 1 hour, 31 minutes	400
SAC	USA	Soyuz MS-09	Jun. 6, 2018	42	196 days, 17 hours, 50 minutes	400

AMC		Soyuz MS-11<br>SpaceX Crew-10	Dec. 3, 2018<br>Mar. 14, 2025	39<br>46	274 days, 4 hours, 35 minutes	420
BM	USA	VSS Unity VF-01<br>Virgin Galactic Unity 22<br>Virgin Galactic Unity 25<br>Galactic 02<br>Galactic 03<br>Galactic 04	Feb. 22, 2019<br>July 11, 2021<br>May 25, 2023<br>Aug. 10. 2023<br>Sept. 08, 2023<br>Oct. 06, 2023	50<br>52<br>54<br>54<br>54<br>54	1 hour, 13 minutes, 15 seconds	86
CK	USA	Soyuz MS-12/13	Mar. 14, 2019	40	328 days, 13 hours, 58 minutes	400
JM	USA/Sweden	Soyuz MS-15	Sep. 25, 2019	42	204 days, 15 hours, 19 minutes	400
SB	USA	Virgin Galactic Unity 22	Jul. 11, 2021	34	14 min 17 seconds	86
WF	USA	Blue Origin NS-16	Jul. 20, 2021	82	~10 minutes	106
SP	USA	Inspiration4	Sep. 16, 2021	51	2 days, 23 hours, 3 minutes	575
HA	USA	Inspiration4	Sep. 16, 2021	30	2 days, 23 hours, 3 minutes	575
YP	Russia	Soyuz MS-19	Oct. 5, 2021	37	11 days, 16 hours, 13 minutes	400
AP	USA	Blue Origin NS-18	Oct. 13, 2021	45	~10 minutes	106
KB	USA	SpaceX Crew-3/Expedition 66	Nov. 10, 2021	34	176 days, 2 hours, 39 minutes	400
LSC	USA	Blue Origin NS-19	Dec. 11, 2021	74	~10 minutes	106
SH	USA	Blue Origin NS-20<br>Blue Origin NS-28	March 31, 2022<br>Nov. 22, 2024	N/A	~10 minutes	106
JW	USA	SpaceX Crew-4	Apr. 27, 2022	34	170 days, 13 hours, 3 minutes	400
KE	USA/Mexico	Blue Origin NS-21	June 04, 2022	27	~10 minutes	106
VOB	USA/UK	Blue Origin NS-22	August 4, 2022	58	~10 minutes	106
SS	Egypt	Blue Origin NS-22	August 4, 2022	29	~10 minutes	106
NAM	USA	SpaceX Crew-5	Oct. 5, 2022	45	157 days, 10 hours, 1 min	420
AK	Russia	SpaceX Crew-5	Oct. 5, 2022	38	157 days, 10 hours, 1 min	420
RB	Saudia Arabia	Axiom Mission 2	May 21, 2023	35	9 days, 5 hours, 27 min	400

JG	USA	Virgin Galactic Unity 25	May 25, 2023	N/A	~10 minutes	86
KL	USA	Galactic 02<br>Galactic 04<br>Galactic 05	Aug. 10, 2023<br>Oct. 06, 2023<br>Nov. 02, 2023	59<br>59<br>59	44 minutes, 21 seconds	86
KS	Antigua and Barbuda	Galactic 02	Aug. 10, 2023	46	~10 minutes	86
AM	Antigua and Barbuda	Galactic 02	Aug. 10, 2023	19	~10 minutes	86
JM	USA	SpaceX Crew-7	Aug. 26, 2023	40	199 days, 2 hours, 20 min	420
LOH	USA	Soyuz MS-24	Sep. 15, 2023	40	203 days, 15 hours, 33 min	420
NS	Pakistan	Galactic 04	Oct. 06, 2023	N/A	~10 minutes	86
KG	USA	Galactic 05	Nov. 02, 2023	34	~10 minutes	86
KM	ITA	Galactic 05	Nov. 02, 2023	67	~10 minutes	86
LB	Ukraine / United States	Galactic 06	Jan. 26, 2024	55	~10 minutes	86
JJE	USA	SpaceX Crew-8	Mar. 4, 2024	54	199 days, 3 hours, 29 min	420
MV	Belarus	Soyuz MS-25/MS-24	Mar. 23, 2024	34	13 days, 18 hours and 41 minutes	400
CS	USA	Blue Origin NS-25	May 19 2024	N/A	~10 minutes	106
NE	UK/Singapore	Blue Origin NS-26	Aug. 29 2024	N/A	~10 minutes	106
KK	USA	Blue Origin NS-26	Aug. 29 2024	21	~10 minutes	106
SG	USA	Polaris Dawn	Sep. 10, 2024	30	4 days, 22 hours, 13 min	575
AM	USA	Polaris Dawn	Sep. 10, 2024	39	4 days, 22 hours, 13 min	575
WH	China	Shenzhou 19	Oct. 29, 2024	34	182 days, 8 hours, 42 min	400
EC	USA	Blue Origin NS-28	Nov. 22, 2024	36	~10 minutes	106
ECH	Australia/USA	Blue Origin NS-30	Feb. 25, 2025	N/A	~10 minutes	106
NA	USA	SpaceX Crew-10	Mar. 14, 2025	39	67 days, 11 hours, 2 minutes	420
JM	Norway/UK	Fram2	Apr. 1, 2025	34	3 days, 14 hours and 32 minutes	450
RR	Germany	Fram2	Apr. 1, 2025	30	3 days, 14 hours and 32 minutes	450
AN	USA	Blue Origin NS-31	Apr. 14, 2025	34	~10 minutes	106
